# Epigenetic silencing of RNF144A expression in breast cancer cells through promoter hypermethylation and MBD4

**DOI:** 10.1002/cam4.1324

**Published:** 2018-02-23

**Authors:** Ye Zhang, Yin‐Long Yang, Fang‐Lin Zhang, Xiao‐Hong Liao, Zhi‐Min Shao, Da‐Qiang Li

**Affiliations:** ^1^ Shanghai Cancer Center and Institutes of Biomedical Sciences Shanghai Medical College Fudan University Shanghai 200032 China; ^2^ Department of Oncology Shanghai Cancer Center Shanghai Medical College Fudan University Shanghai 200032 China; ^3^ Cancer Institute Shanghai Cancer Center Shanghai Medical College Fudan University Shanghai 200032 China; ^4^ Department of Breast Surgery Shanghai Cancer Center Shanghai Medical College Fudan University Shanghai 200032 China; ^5^ Key Laboratory of Breast Cancer in Shanghai Shanghai Medical College Fudan University Shanghai 200032 China

**Keywords:** Breast cancer, DNA methylation, epigenetic regulation, MBD4, RNF144A

## Abstract

Emerging evidence shows that ring finger protein 144A (RNF144A), a poorly characterized member of the Ring‐between‐Ring (RBR) family of E3 ubiquitin ligases, is a potential tumor suppressor gene. However, its regulatory mechanism in breast cancer remains undefined. Here, we report that *RNF144A* promoter contains a putative CpG island and the methylation levels of *RNF144A* promoter are higher in primary breast tumors than those in normal breast tissues. Consistently, *RNF144A* promoter methylation levels are associated with its transcriptional silencing in breast cancer cells, and treatment with DNA methylation inhibitor 5‐Aza‐2‐deoxycytidine (AZA) reactivates RNF144A expression in cells with *RNF144A* promoter hypermethylation. Furthermore, genetic knockdown or pharmacological inhibition of endogenous methyl‐CpG‐binding domain 4 (MBD4) results in increased RNF144A expression. These findings suggest that RNF144A is epigenetically silenced in breast cancer cells by promoter hypermethylation and MBD4.

## Introduction

Ring finger protein 144A (RNF144A) belongs to the Ring‐between‐Ring (RBR) family of E3 ubiquitin ligases and is highly conserved in eukaryotes 1. The members of the RBR protein family contain three functional domains, including two separated RING finger domains and an in‐between ring (IBR) domain 1. The best known of the RBR family of E3 protein ligases is Parkin 2, which has a prominent role in the manifestation of early‐onset Parkinson's disease^3^, ^4^. Emerging evidence shows that RNF144A promotes apoptosis in response to DNA damage through targeting DNA‐dependent protein kinase (DNA‐PK) for ubiquitination and proteasomal degradation 5. Our recent studies demonstrated that induced expression of RNF144A decreases the sensitivity of breast cancer cells to poly(ADP‐ribose) polymerase (PARP) inhibitor olaparib through targeting DNA repair protein PARP1 for ubiquitination and subsequent proteasomal degradation^6^. However, the regulatory mechanism of RNF144A in breast cancer remains unknown.

Epigenetic silencing of tumor suppressor genes by DNA methylation has emerged as one of the pivotal alterations in cancer development and progression 7, 8. In humans, DNA methylation mainly occurs in high‐CpG dinucleotide sequences, which are defined as CpG islands 9, 10. Given that DNA methylation represses the binding of transcription factors to promoters or establishes a repressive structure of chromatin, high methylation of a coding gene promoter induces the silence of the genes by suppression of its transcription 10, 11. Typically, the promoter regions of oncogenes are hypomethylated, whereas tumor suppressor genes are hypermethylated 12. Accumulating evidence shows that proteins of the methyl‐CpG‐binding domain (MBD) family are primary candidates for the readout of DNA methylation as they recruit the enzymatic machinery to establish silent chromatin 8, 13. Methyl‐CpG domain protein 4 (MBD4), a MBD family protein, binds specifically to methylated DNA through a conserved MBD domain and represses transcription of genes with methylated promoters 14, 15. For instance, MBD4 has been shown to bind to the hypermethylated promoters in tumor suppressor genes *p16INK4a* and *hMLH1* and repress their transcription 14. More recently, it was reported that the RON receptor tyrosine kinase upregulates MBD4 through activation of the phosphoinositide 3‐kinase (PI3K) pathway to promote breast cancer metastasis 16. However, the precise role of MBD4 in the regulation of gene transcription remains elusive.

In this study, we provide evidence for the first time that RNF144A is epigenetically silenced by promoter hypermethylation and MBD4. These findings provide new mechanistic insights into the regulatory mechanisms of RNF144A in breast cancer cells.

## Materials and Methods

### Cell lines and cell culture

The well‐characterized breast cancer cell lines T47D, MCF7, MDA‐MB‐453, BT20, MDA‐MB‐231 and the immortalized normal breast cell line HBL100 were purchased from the Type Culture Collection of Chinese Academy of Sciences (Shanghai, China). Cells were maintained in DMEM medium (Cellgro, Manassas, VA) supplemented with 10% fetal bovine serum (Gibco, Carlsbad, CA), 50 U/mL penicillin and 50 *μ*g/mL streptomycin (BasalMedia, Shanghai, China).

### Patient samples

Total 30 matched primary breast cancer tissues and adjacent noncancerous breast tissues were obtained from breast cancer patients who were diagnosed with invasive breast ductal carcinoma and underwent surgery at Shanghai Cancer Center, Fudan University. Adjacent normal tissues were at least 2 cm away from the tumor margins and histologically confirmed as cancer free. Tissue samples were collected with written informed consent from patients under institutional review board‐approved protocol. Samples were immediately frozen in liquid nitrogen and split into two halves. One half of those samples were used for qPCR analysis of RNF144A mRNA levels, and the other half of them were used for pyrosequencing analysis of *RNF144A* promoter methylation levels. Characterization of clinicopathological features of these breast cancer patients was described in Table 1.

**Table 1:** Characterization of clinicopathological features of 30 primary breast cancer patients

Clinicopathological features	No. of patients	Percentage (%)
Age (31–77 years, 51.5 ± 10.12 years)		
≦50	20	66.7
>50	10	33.3
Menopausal status		
Premenopausal	21	70
Postmenopausal	9	30
Tumor size		
≦2 cm	16	53.3
>2 cm	14	46.7
Lymph node status		
Negative	15	50
Positive	15	50
Grade		
I‐II	20	66.7
III	10	33.3
ER status		
Negative	21	70
Positive	9	30
PR status		
Negative	22	73.3
Positive	8	26.7
HER2 status		
Negative	20	66.7
Positive	10	33.3
Clinical stage (TNM)		
I‐II	23	76.7
III	7	23.3

### Pyrosequencing of RNF144A methylation

Genomic DNA from cells and tissues was extracted using the QIAamp DNA Mini Kit (Qiagen, Valencia, CA), and then 1 *μ*g of extracted DNA was subjected to bisulfite modification using the EZ DNA Methylation‐Gold Kit (Zymo Research, Irvine, CA) according to the manufacturer's instructions. Bisulfite treated‐genomic DNA was used as a template to amplify a product of 421 bp by a NEST PCR. The first product (566 bp) was amplified using the PCR1 primer set, and the second product (421 bp) was amplified using the first product as the template and the PCR2 primer set. Then the second product was used to detect two specific regions of *RNF144A* promoter (R1: −131 to −72; R2: +24 to +72) for quantitative DNA methylation analysis by pyrosequencing. This technology allows the quantification of the degree of methylation at each CG site through the calculation of the ratio between T and C 17. The primers were designed by Methyl Primer Express v1.0 software, and related sequences were as follows:

- The shared 5′‐Biotin‐tag reveres primer: TCACTCTACCTAAACCTACTCRTCC;
- PCR1 forward primer: TTTYGGTGTAGGAATTAGGGGAGTG;
- PCR2 forward primer: GGAGGTTATTAAAGTAGGAATAGTA;
- Sequence 1 forward primer: GTAGGAATAGTATTTTTATGTTAGYGTGTA
- Sequence 2 forward primer: GAGTTTTTYGYGTGTTYGGTTTTTGTG.

### Quantitative real‐time PCR

Total RNA was extracted from cell lines or frozen patient samples using TRIzol reagent (Invitrogen, Carlsbad, CA), and the first‐strand cDNAs were generated using PrimeScript RT master mix (Takara, Dalian, China) according to the manufacturer's protocol. Quantitative real‐time PCR (qPCR) was performed using FS Universal SYBR Green Master kit (Roche, Shanghai, China) on a Mastercycler ep realplex machine (Eppendorf, Germany). Cycling conditions were one cycle at 95°C for 10 minutes followed by 40 cycles of 95°C for 10 sec, 60°C for 5 sec, and 72°C for 10 sec. The fold change in the expression levels of RNF144A mRNA relative to housekeeping gene *GAPDH* was calculated based on the threshold cycle (C_t_) as 2^−Δ(ΔCt)^ 18, where ΔC_t _= C_t_ (RNF144A) − C_t_ (GAPDH). The primer sequences were as follows: human RNF144A forward: CCACCTACAGGAGAACGAG, reverse: TCCGACAGGGATCAAACA; human GAPDH forward: CGAGATCCCTCCAAAATCAA, reverse: TTCACACCCATGACGAACAT.

### Drug administration

For treatment with demethylating agent 5‐Aza‐2′‐deoxycytidine (AZA; Sigma‐Aldrich, St. Louis, MO), 2 × 10^5^ cells were seeded in the six‐well plates, allowed to attach for 24 h, and then treated with increasing doses of AZA for 4 d. The medium containing AZA was replaced every 24 h. For treatment with selective PI3K inhibitor wortmannin (Selleck Chemicals, Houston, TX), 1.5 × 10^6^ cells were seeded in the six‐well plates, allowed to attach for 24 h, and then treated with 200 nmol/L Wortmannin as reported previously 19, 20, 21 and harvested at indicated time intervals.

### Western blot analysis

Total cell proteins were extracted using modified RIPA buffer (50 mmol/L Tris–HCl, pH 7.4, 150 mmol/L NaCl, 1% NP‐40, 0.25% sodium deoxycholate, and 1 mmol/L EDTA) containing protease (Roche) and phosphatase (Selleck Chemicals) inhibitors. The soluble protein samples were collected by centrifuged at 14,000 × rpm for 20 min in 4°C. Proteins were quantified using the BCA kit (Yeasen, Shanghai, China). Proteins were isolated on 10% SDS‐PAGE gel and then transferred to PVDF membrane (Millipore, Billerica, MA), followed by antibody detection using enhanced chemiluminescence reagents (Yeasen). The dilution of specific antibodies for human RNF144A (LS‐C162648, LifeSpan Biosciences, Seattle, WA) was 1:1000, for human MBD4 (sc‐365974, Santa Cruz Biotechnology, Santa Cruz, CA) was 1:1000, and for human vinculin (V9131, Sigma‐Aldrich) was 1:5000. The second antibodies were goat anti‐mouse IgG‐HRP (7074V, Cell Signaling Technology, Danvers, MA) and goat anti‐rabbit IgG‐HRP (7076V; Cell Signaling Technology), and the dilution of the second antibodies was 1:5000.

### Knockdown of endogenous MBD4 by short hairpin RNAs

Short hairpin RNAs (shRNAs) targeting human MBD4 (shMBD4) and negative control shRNAs (shNC) were purchased from Origene (Rockville, MD). For lentiviral infection experiments, HEK293T cells were transfected with lentivirus expression vectors and packaging plasmid mix using Teng‐fect DNA transfection reagents (Tengyi Biotech, Shanghai, China). Media with progeny virus was collected after 48 h of transfection and filtered with 0.45‐*μ*m filters (Millipore). To generate MBD4‐knocked down stable cell lines, MDA‐MB‐231 cells were infected with lentiviral supernatants diluted 1:1 with culture medium in the presence of 8 *μ*g/mL of polybrene (Sigma‐Aldrich). After 24 h of infection, cells were selected with 2 *μ*g/mL puromycin (Cayman Chemical, Ann Arbor, MI) for 1 week and then passaged before use.

### Data mining

The genetic alternations of RNF144A gene in breast cancer were analyzed using the cBioPortal for Cancer Genomics 22, 23. The presence of any putative CpG islands at *RNF144A* promoter (−1000 to +100 relative to the transcription start site) was analyzed using the MethPrimer 24 and the CpG plot 25 programs. *RNF144A* promoter methylation intensity in normal and breast cancer tissues was analyzed using The Cancer Methylome System 26.

### Statistical analysis

The statistical data were analyzed by SPSS software version 22. Student's *t*‐test was used to analyze data between two groups. Results are presented as mean ± SD. *P* value <0.05 was considered as significant difference.

## Results

### *RNF144A* promoter contains a putative CpG island

**Figure 1:**
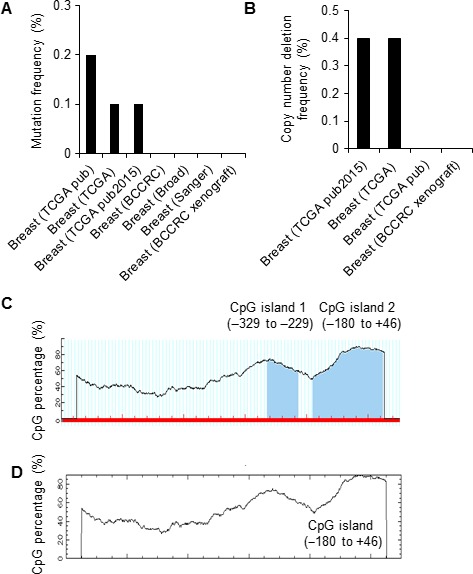
*RNF144A* promoter contains a putative CpG island. (A‐B) Alteration frequency of *RNF144A* gene mutation (A) and copy number deletion (B) in human breast cancer at the cBioPortal for Cancer Genomics. (C‐D) Predication of CpG islands at the promoter region of *RNF144A* gene (−1000 to +100) using the MethPrimer software (C) and the CpG plot program (D).

Breast cancer is characterized by both genetic and epigenetic alterations 27. To elucidate the underlying mechanisms for RNF144A in breast cancer, we first analyzed the possible genetic alternations of *RNF144A* gene in breast cancer at the cBioPortal for Cancer Genomics 22, 23. Interestingly, we found that the alteration frequency for *RNF144A* gene mutation (Fig. 1A) and copy number deletion (Fig. 1B) in human breast cancer is no more than 0.2% and 0.4%, respectively, suggesting that those two types of genetic alterations may not be the major cause of RNF144A deregulation in breast cancer.

In addition to genetic alterations, epigenetic silencing is another key mechanism for inactivation of tumor suppressor genes during cancer development and progression 27. To date, DNA methylation is the most studied epigenetic event in cancer 28. To determine whether aberrant promoter hypermethylation underlines altered RNF144A expression in breast cancer, we next analyzed the presence of any putative CpG islands on its promoter (−1000 to +100 relative to the transcription start site) using the MethPrimer program 24. Typical CpG islands were defined as regions of DNA with a high G+C content (greater than 50%) and observed CpG/expected CpG ratio of greater or equal to 0.6 29. Following the criteria with island size >100 bp, we found two CpG islands present in *RNF144A* promoter, located at −329 to −229 and −180 to +46, respectively (Fig. 1C). Another analysis of *RNF144A* promoter (−1000 to +100) using the CpG plot program 25 demonstrated the presence of one putative CpG island, located at −180 to +46 (Fig. 1D). This program defines a CpG island as ≥200 bp with ≥50% C+G content and ≥0.6 CpG observed/CpG expected. Together, two independent bioinformatic analyses demonstrated the presence of one putative CpG island in *RNF144A* promoter (−180 to +46). Given that the majority of CpG islands in the human genome are heavily methylated 30, these results indicate that *RNF144A* promoter might be methylated in breast cancer.

### The methylation levels of *RNF144A* promoter are increased in breast tumors

**Figure 2:**
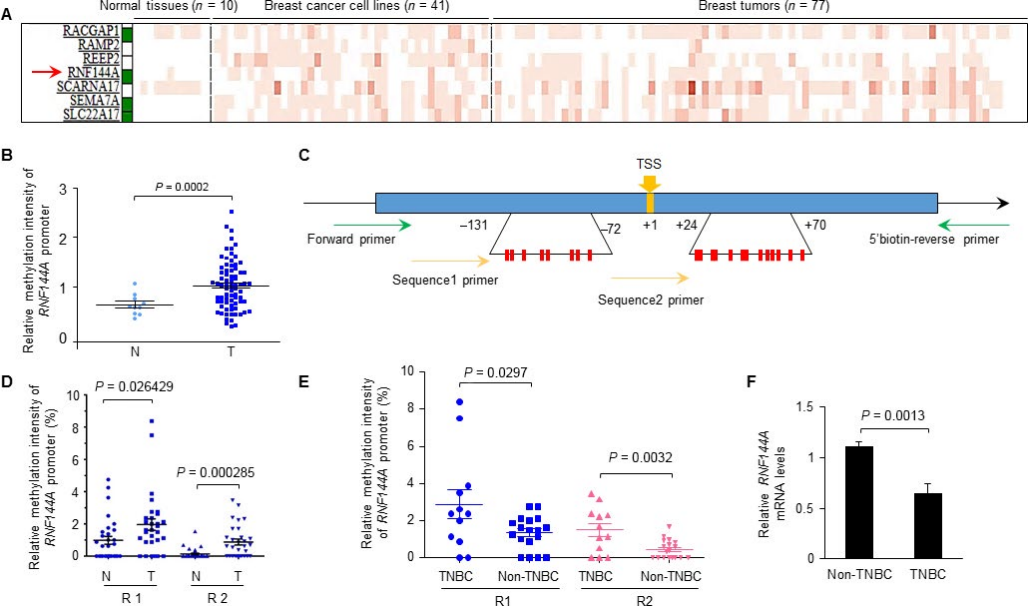
The methylation levels of *RNF144A* promoter are increased in breast tumors. (A‐B) Analysis of the methylation levels of *RNF144A* promoter in normal and breast cancer tissues at The Cancer Methylome System (A). This database includes 10 normal breast samples, 41 breast cancer cell lines, and 77 breast tumor samples. The red arrow stands for methylation pattern of *RNF144A* gene. Methylation intensities for promoter regions of genes are shown using a red gradient heatmap. A white/green box on the side of gene symbol shows the promoter regions of this particular gene with or without CpG island(s). Gene promoter containing CpG island (s) is indicated in green 26. Corresponding quantitative results are shown in B. N indicates normal breast tissues (*n *= 10); T indicates breast tumors (*n *= 77). (C) Schematic diagram of pyrosequencing of *RNF144A* promoter regions. The transcription starting site (TSS) was marked as +1. The promoter regions of *RNF144A* which contain the putative CpG island (R1: −131 to −72; R2: +24 to +70) were amplified by PCR and then subjected to pyrosequencing. (D) Pyrosequencing analysis of the methylation levels at two separate regions (R) (R1 and R2) of the *RNF144A* promoter in 30 pairs of breast cancer specimens and matched adjacent noncancerous breast tissues. N indicates normal breast tissues; T indicates breast tumors. (E) Analysis of DNA methylation levels in regions 1 (R1) and 2 (R2) of *RNF144A* promoter in TNBC (*n *= 12) and non‐TNBC (*n *= 18) breast cancer specimens from 30 breast cancer patients as mentioned above in D. (F) qPCR analysis of RNF144A mRNA levels in TNBC (*n *= 12) and non‐TNBC (*n *= 18) breast cancer specimens from 30 breast cancer patients.

To determine whether DNA methylation levels in *RNF144A* promoter are indeed altered in breast cancer, we analyzed *RNF144A* promoter methylation intensity in normal and breast cancer tissues at The Cancer Methylome System 26. This system is a web‐based database application designed for the visualization, comparison, and statistical analysis of human cancer‐specific DNA methylation 26. Indeed, we found significantly higher methylation levels of *RNF144A* promoter in primary breast tumors relative to normal breast tissues (Fig. 2A and B).

To confirm these results, we next analyzed the methylation levels in the CpG islands of RNF144A in 30 pairs of breast cancer specimens and matched adjacent noncancerous breast tissues by pyrosequencing 17. To this end, the *RNF144A* promoter region that contains the predicted CpG islands (−180 to +46) was amplified by PCR and then subjected to pyrosequencing for analyzing the relative methylation levels of *RNF144A* promoter in two separate regions (R) (R1 to R2: −131 to −72 and +24 to +70, respectively) (Fig. 2C). Results showed that the relative methylation levels of *RNF144A* promoter at the regions R1 and R2 were significantly higher in breast tumor samples relative to matched normal breast tissues (Fig. 2D). To further analyze whether the alternation of methylation levels of *RNF144A* promoter is associated with different molecular subtypes of breast cancer, we compared the methylation levels of *RNF144A* promoter at the R1 and R2 regions in triple‐negative breast cancer (TNBC, *n *= 12) with those in non‐TNBC (*n *= 18) in our patient cohort. As shown in Figure 2E, the relative methylation levels of *RNF144A* promoter at the R1 and R2 regions were significantly higher in TNBC relative to non‐TNBC tumor samples. Consistently, the mRNA levels of RNF144A were lower in TNBC (*n *= 12) as compared with those in non‐TNBC samples (*n *= 18; Fig. 2F, *p* = 0.0013).

### Promoter hypermethylation is associated with transcriptional silencing of RNF144A in breast cancer cells

**Figure 3:**
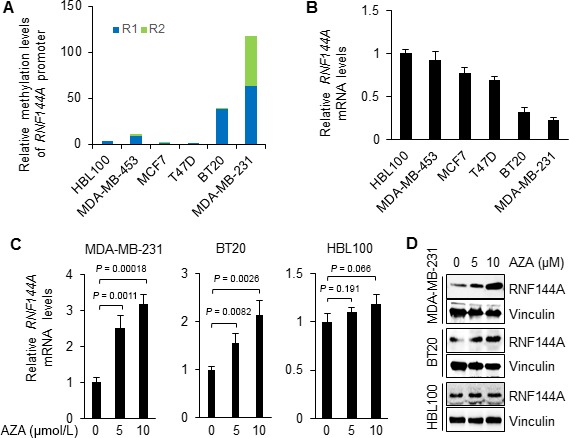
Promoter hypermethylation is involved in transcriptional silencing of RNF144A in breast cancer cells. (A) The methylation levels of *RNF144A* promoter in normal epithelial cell line HBL100 and five representative breast cancer cell lines. (B) qPCR analysis of RNF144A mRNA levels in HBL100 and five breast cancer cell lines. Data are shown as mean ± SD. (C–D) Cells were treated with DNA methylation inhibitor AZA at the indicated doses for 4 d and subjected to qPCR (C) and immunoblotting (D) analysis of RNF144A mRNA and protein levels, respectively. Data are presented as mean ± SD in C.

We next analyzed the DNA methylation levels of *RNF144A* promoter at the R1 and R2 regions by pyrosequencing in normal mammary epithelial cell line HBL100 and 5 representative breast cancer cell lines, including MCF7, T47D (luminal‐type), MDA‐MB‐453 (HER2‐positive), BT20, and MDA‐MB‐231 (TNBC). Consistent with the above results, high methylation levels of *RNF144A* promoter were observed in triple‐negative BT20 and MDA‐MB‐231 cell lines (Fig. 3A). In contrast, the methylation levels were relatively low in normal HBL100 and luminal‐type and HER2‐positive cell lines (Fig. 3A). qPCR analysis showed an inverse association between promoter methylation and mRNA expression levels of RNA144A (Fig. 3B). These results indicate that hypermethylation of CpG islands at the *RNF144A* promoter may underlie RNF144A downregulation in breast cancer.

To directly analyze whether expression of RNF144A is regulated by promoter methylation, we next examined the effects of DNA methylation inhibitor AZA on the reactivation of RNF144A in breast cancer cells by qPCR and immunoblotting analyses. qPCR analysis results showed that treatment of MDA‐MB‐231 and BT20 cells, in which *RNF144A* promoter was hypermethylated, with AZA significantly reactivated RNF144A expression in a dose‐dependent manner (Fig. 3C, left and middle panels, respectively). In contrast, incubation of HBL100 cells, in which *RNF144A* promoter was hypomethylated, with the same doses of AZA did not significantly alter the mRNA levels of RNF144A (Fig. 3C, right panel). Consistently, immunoblotting analysis demonstrated that protein levels of RNF144A were increased in MDA‐MB‐231 and BT20, but not in HBL100, cells following AZA treatment (Fig. 3D). These results suggest that DNA methylation is involved in epigenetic regulation of RNF144A expression in breast cancer cells.

### MBD4 transcriptionally represses RNF144A expression in breast cancer cells

**Figure 4:**
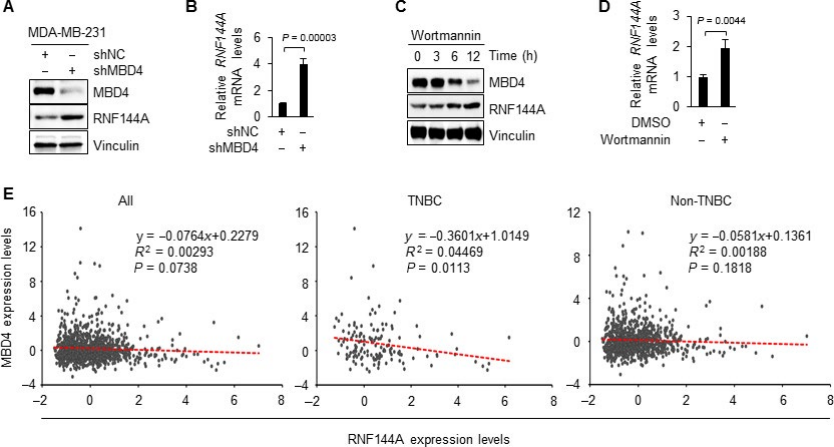
MBD4 transcriptionally represses RNF144A expression in breast cancer cells. (A‐B) MDA‐MB‐231 cells stably expressing shNC and shMBD4 were subjected to immunoblotting (A) and qPCR (B) analysis of RNF144A expression levels. (C) MDA‐MB‐231 cells were treated with DMSO or 200 *nmol/L* Wortmannin for the indicated times and then subjected to immunoblotting analysis with the indicated antibodies. (D) MDA‐MB‐231 cells were treated with DMSO or 200 nmol/L Wortmannin for 24 h and analyzed by qPCR analysis of RNF144A mRNA levels. (E) The correlation between the expression levels of MBD4 and RNF144A in breast cancer patients in TCGA database.

Previous studies have documented that MBD4 binds specifically to methylated DNA to repress transcription of genes with methylated promoters 14, 15 and the PI3K kinase upregulates MBD4 expression 16. We next examined whether MBD4 is involved in the regulation of RNF144A in breast cancer cells. To this end, we knocked down endogenous MBD4 in MDA‐MB‐231 cells using specific shRNAs targeting MBD4 (shMBD4). Immunoblotting and qPCR analyses showed that depletion of MBD4 resulted in an upregulation of RNF144A protein and mRNA levels (Fig. 4A and B). Consistently, we found that protein (Fig. 4C) and mRNA (Fig. 4D) levels of RNF144A were upregulated following inhibition of MBD4 by selective PI3K inhibitor Wortmannin 16. These results suggest that MBD4 transcriptionally represses RNF144A expression in breast cancer cells.

Following these observations, we next examined whether there is a negative correlation between the expression levels of MBD4 and RNF144A in breast cancer in The Cancer Genome Atlas (TCGA) database. As shown in Figure 4E, there is a marginal correlation between the expression levels of MBD4 and RNF144A in all of patients with breast cancer (*n *= 1093, *p *=* *0.0738). In particular, the expression levels of RNF144A are negatively associated with those of MBD4 in TNBC patients (*n *= 143, *p *=* *0.0113) but not in non‐TNBC patients (*n *= 950, *p *=* *0.1818). These results suggest that MBD4 mainly mediates transcriptional repression of RNF144A expression in TNBC patients. Moreover, these results are consistent with the findings that the relative methylation levels of *RNF144A* promoter were significantly higher in TNBC relative to non‐TNBC tumor samples (Fig. 2E).

## Discussion

DNA hypermethylation is a well‐recognized mechanism underlying gene silencing events in cancer development and progression 31. Tumor suppressors are frequently inactivated by DNA methylation at CpG islands of gene promoters 32. For instance, breast cancer progression is often accompanied with hypermethylation on the promoter region of tumor suppressive genes, such as breast cancer metastasis suppressor 1 (*BRMS1*) 33 and *E‐cadherin* 34. Clinical evidence also suggests that patients with hypermethylation on the promoter region of tumor suppressive genes, such as Ras association domain family member 1 (*RASSF1A*) 35, *BRMS1* 33, and breast cancer type 1 susceptibility gene (*BRCA1*) 36, 37, 38, are associated with poor prognosis.

RNF144A is a poorly characterized member of the RBR family of E3 ubiquitin ligases 39. Emerging evidence shows that RNF144A is involved in p53‐mediated apoptosis during DNA damage 5. In addition, our recent study demonstrated that RNF144A decreases cellular sensitivity to PARP inhibitor olaparib in a xenograft mouse model through targeting PARP1 for ubiquitination and degradation^6^. These results indicate that RNF144A may exert tumor suppressive activities in human cancers. In the present study, we provide evidence for the first time that promoter hypermethylation is involved in transcriptional repression of RNF144A in breast cancer cells. There are several lines of evidence supporting this notion. First, *RNF144A* promoter contains a putative CpG island, and the methylation levels of *RNF144A* promoter are higher in primary breast tumors than those in normal breast tissues (Figs 1 and 2). Second, pyrosequencing and qPCR analysis showed that the methylation levels of *RNF144A* promoter are negatively associated with its mRNA expression levels in breast cancer cells (Figs 2 and 3). Of note, we found that TNBC tumors and TNBC breast cancer cell lines showed the relative high methylation levels on the promoter region of *RNF144A*. The results indicate the DNA methylation regulation for RNF144A varies in the different subtypes of breast cancer. Third, treatment of breast cancer cells with DNA methylation inhibitor AZA effectively restored the mRNA and protein expression of endogenous RNF144A (Fig. 3). Fourth, previous studies have shown that MBD4 binds to methylated promoters of multiple tumor suppressor genes, such as *CDKN1A/p21* 40, *p16(INK4a)*, and *hMLH1* 14 and transcriptionally represses gene expression. Evidence from pharmacological and genetic modulation of MBD4 demonstrated that MBD4 is involved in RNF144A expression in breast cancer cells (Fig. 4). In support of our findings, Cunha et al. 16 using RNA‐sequencing and microarray gene expression profiling demonstrated that *RNF144A* is one of 192 genes that are regulated by MBD4 through aberrant DNA methylation in human breast cancer cells. However, we cannot rule out the possibility that inhibition of MBD4 resulting in increased expression of RNF144A could be mediated by DNA methylation‐independent pathways. In addition, a possible limitation of the present study was the relatively small sample size of patient samples and breast cancer cell lines used in the *RNA144A* promoter methylation analyses. Clearly, further studies are warranted to confirm the relationship between DNA methylation and RNF144A expression levels in a large size of breast tumor samples.

In summary, findings presented here show that reduced RNF144A expression in breast cancer cells is regulated by promoter hypermethylation and MBD4 in breast cancer cells. Together with our recent studies and others 5, 6, the clinical use of demethylating agents such as AZA and PI3K inhibitor Wortmannin could block breast cancer development and progression through reactivation of tumor suppressor RNA144A expression.

## Conflict of Interest

The authors declare no potential conflict of interests.
